# Is metaphor a natural kind?

**DOI:** 10.3389/fpsyg.2024.1381821

**Published:** 2024-03-25

**Authors:** Stefana Garello, Marco Carapezza

**Affiliations:** ^1^Unit of Neuroscience, Department of Medicine and Surgery, University of Parma, Parma, Italy; ^2^Humanistic Sciences Department, University of Palermo, Palermo, Italy

**Keywords:** metaphor, metaphor studies, natural kind, cognitive linguistics, pragmatics, figurative language

## Abstract

In Metaphor Studies, metaphor is considered as a “form of understanding one thing in terms of something else.” It is assumed that, despite their differences, metaphors share many properties and that a theory of metaphor should capture these essential properties. In short, it is assumed that metaphor is a natural kind. We call this view the Natural Kind Assumption. In this paper, we will challenge it and show that metaphor is not a natural kind. Finally, we will discuss the main philosophical consequences of this view.

## 1 Introduction

Over the last five decades, the number of theories, hypotheses, evidence and data on metaphor has grown considerably. The extensive work on metaphor makes Booth’s prediction true (1979, 49), for which “by the year 2039, there will be more students on metaphor on Earth than people”.

The interest in metaphors has led to the development of a fruitful field of research at the interface of philosophy, linguistics and cognitive science, known as Metaphor Studies. Research in this field differ considerably in their approaches and definitions. Despite these differences, however, there is a general tendency to accept that the use of metaphor is a “form of understanding one thing in terms of something else” (1). From this perspective, metaphors are assumed to represent a homogeneous class of phenomena and therefore share a large number of properties. The aim of a theory of metaphor is to grasp these common properties.

This view of metaphor, which runs like a common thread through all metaphor theories of the last fifty years, essentially assumes that metaphors constitute a natural kind. In other words, metaphors represent a category of entities for which numerous inductive generalizations can be developed. Despite the differences between metaphors, it is essential to find generalizations about the members of this category. Using Boyd’s terminology (1990), we will define this perspective on metaphor as the Natural Kind Assumption (henceforth NKA). In contrast to the prevailing acceptance of the NKA, this paper attempts to challenge it. Contrary to the assumptions of Metaphor Studies, we argue that metaphors do not constitute a homogeneous category for which numerous inductive generalizations can be made. In essence, we assume that metaphor is not a natural kind. We will explore the main implications of this hypothesis for a theory of language, especially in reference to metaphor research.

Specifically, first we will examine what a natural kind is. By showing the variety of theories and data concerning metaphor, we will argue that metaphor cannot be a natural kind. Consequently, the term “metaphor” is not a natural kind term. It denotes different phenomena that are too heterogeneous to be considered as a single natural kind. Instead, “metaphor” is a complex, theory-dependent philosophical notion. Finally, we will discuss the main consequences of this hypothesis for Metaphor Studies.

## 2 What is a natural kind?

A Natural Kind identifies classes that reflect the structure of the natural world, exist independently of our conceptualization and around which relevant inductive generalizations can be developed (Machery, 2005). In particular, the literature on natural kinds assumes that there are two kinds of classes (see Bird, 2022): those for which inductive generalizations can be formulated (e.g., the class of gold, which seems to exist without our conscious categorization), and those for which few or no generalizations can be made (e.g., objects weighing more than 50 kg). There seems to be little scientific interest in anything that weighs more than 50 kg, other than the fact that we perceive it as heavier than 50 kg. While the elements that belong to the class of gold share a common and non-accidental physical property, the elements that belong to things that weigh more than 50 kg share a property in a completely accidental way, and therefore there is nothing that allows us to identify properties within the class of “things that weigh more than 50 kg” that distinguish them from other classes. In Boyd ’s (1990, 1991) words: “A class of C entities is a Natural Kind if and only if there is a large set of scientifically relevant properties such that C is the maximal class whose members tend to share these properties because of some causal mechanisms.”

A natural kind is thus a category for which numerous generalizations can be developed. Its members are expected to systematically possess a considerable number of relevant properties that are not accidental. It is important to emphasize that these properties are not accidental, as there is at least one causal mechanism that explains why the members of this category tend to exhibit these properties. It is also important to note that this category should not be a subset of a larger class for which the same generalizations could be formulated.

Therefore, the following conditions must be met for a class to be considered a Natural Kind:

– There is an essential and not accidental set of properties common to these phenomena.
– The presence of these properties is an essential prerequisite for defining an element as belonging to this class.
– There must be specific properties that distinguish the elements of this class from other phenomena.

However, in reality, there is another kind of class, namely one that deals with abstract entities that exist on a different ontological level than the physical world. Think, for example, of scientific constructs and theoretical objects, entities whose empirical verification in the world is quite problematic. Often, however, this third class of objects is studied like the first class, i.e., as empirically verifiable entities in the world that share essential physical properties in a non-accidental way. In short, this third class is treated as if the elements of which it is composed were natural kinds. But can this third class of objects be said to constitute a natural kind? Some confusion seems to arise between the ontological levels of reality, where theoretical constructs are equated with chemical and empirical elements. However, in the history of philosophy, especially since the second half of the Twentieth Century, various authors have raised the question of whether it is possible to treat our theoretical constructs as natural kinds. This was the case with Davidson (1985), who argued that language is not a natural kind. More recently, Machery (2005) has raised the problem of concepts: can we say that concepts, which cognitive science holds to be the primary elements of our minds, are natural kinds? Machery’s answer is no, concepts are not natural kinds, and the term *concept* “is inappropriate to carve human beings’ mental representations at their joints, if one aims at formulating empirically relevant inductive generalizations about human minds” (Machery, 2005, 2). Similarly, Lohr (2023) and Taylor (2020) also pose this question for the notions of “concreteness” and “emotions”. While Lohr argues that concreteness and concrete concepts are not natural kinds, Taylor argues that “it is indeterminate whether concepts and emotions are natural kinds. They are neither determinately natural kinds, nor determinately non-natural kinds” (Taylor, 2020, 2073). Leezenberg (2007) and shortly thereafter Sperber and Wilson (2008) note that metaphors have also been treated as natural kinds for centuries, especially since Lakoff and Johnson’s Conceptual Metaphor Theory (1980), and argue that this treatment is inappropriate. In this regard, the authors write:

There is no mechanism specific to metaphors, no interesting generalization that applies only to them. In other terms, linguistic metaphors are not a natural kind, and ‘metaphor’ is not a theoretically important notion in the study of verbal communication” (Sperber and Wilson, 2008, 97).

In the case of Sperber and Wilson (2008), there is even a form of deflationism and eliminativism of the term “metaphor”. According to the authors, an utterance can be considered literal or metaphorical depending on the context: if the utterance “the water is boiling” is uttered in relation to a pot of boiling water, it is interpreted literally; if it is uttered in front of very hot but not boiling water, we have a hyperbolic interpretation; if, on the other hand, the utterance is made in the midst of a tense political situation, it receives a metaphorical interpretation. In all these cases, the same processes of contextual modulation of meaning are at work. For this reason, Sperber and Wilson argue in a radical way that metaphor is not a natural kind and that the term “metaphor” is not an interesting theoretical notion for the study of human language.

In the next section we will discuss whether metaphor fulfills the requirements to be considered a natural kind, and therefore whether the question “what is a metaphor?” should be treated as a question about entities that constitute a natural kind (e.g., “what is the amygdala?”), or whether such a question should otherwise be considered a philosophical question and the notion of metaphor should be treated as a multifaceted theoretical construct.

## 3 Is metaphor a natural kind?

We can call the perspective that claims that metaphors constitute a natural kind the Natural Kind Assumption (NKA). This assumption holds that metaphor is a unified and homogeneous empirical phenomenon and that, despite superficial differences, there are several not accidental and shared properties between the member of this category. A theory of metaphor should aim to capture these properties. This assumption, which is widely accepted in metaphor research, is based on three premises:

(1) Metaphor, despite their differences, share not accidental common features. “Metaphor” represents a class of homogeneous phenomena with common properties.
(2) The presence of these properties is the essential precondition for defining an element as metaphorical.
(3) Metaphors have specific properties that distinguish them from other types of phenomena.

Despite the widespread support for the NKA, we will now challenge this perspective. We will emphasize that metaphors are not a unitary kind for which generalizations can be made.

### 3.1 The NKA leads to the antinomies of metaphor

There is some confusion to be observed in the context of Metaphor Studies, and it seems to be related to the NKA. In particular, it is possible to identify three key issues around which Metaphor Studies revolve and to discuss contrasting theories and empirical evidence on this interesting and complex phenomenon:

(1) Is metaphor a matter of style or of thought?
(2) What is and how is the meaning of a metaphor constructed?
(3) What is the relationship between literal and metaphorical meaning?

It is important to clarify that it is not possible to expect precise and unambiguous answers to these questions. No theory and no field of research seems to be able to grasp the metaphorical phenomenon comprehensively and accurately. Each theory and each answer to these questions emphasizes certain aspects of the metaphorical phenomenon while neglecting other equally important aspects. Similarly, each theory and question seem to have valid reasons supporting both the claim and its opposite, leading to what can be termed “antinomies” in metaphor research – contradictory hypotheses, all supported by data, to which a precise answer is difficult, if not impossible. Given the compelling arguments in favor of both the thesis and antithesis of each antinomy, it is a great challenge to take a definitive stand on each antinomy.

Regarding the first antinomy – is metaphor a matter of style or of thought? – against a tradition that views metaphor as a figure of style (see Johnson, 1981) authors such as Tesauro (1670), Vico (1744/1977), Nietzsche (1873), Richards (1936/95), Blumenberg (1960/2010), Black (1962), Ricoeur (1975), Kittay (1987) and, finally, Lakoff and Johnson (1980) argue that metaphor plays an essential and peculiar role in our cognition. It is a mechanism, rather than an utterance, which enables us to “see the similar in the dissimilar” and thereby create new knowledge. According to Lakoff and Johnson, metaphor represents the way in which our conceptual system is constructed through mappings between concrete and abstract concepts.

The second antinomy reveals a contradiction with regard to what is meant by the term “metaphorical meaning” and how it is constructed. According to some hypotheses, metaphorical meaning is an independent, secondary meaning that is constructed by inferential processes in relation to propositions (Grice, 1975; Searle, 1979; Sperber and Wilson, 2008). We will refer to this group of hypotheses as Proposition Theories of metaphor (hereafter PTM). According to other authors, however, metaphor has no meaning of its own, but it means what words mean in their literal sense (Davidson, 1978; Lepore and Stone, 2015) since the peculiarities of metaphors lie on its perlocutionary effects, that are non-propositional effects. As Rorty (1987) likes to say, metaphor is an “unfamiliar noise” like the song of an unfamiliar bird. We refer to this hypothesis as the Image Theories of metaphor (hereafter ITM).

Finally, the third antinomy can be seen as an internal debate within PTM about the importance of literal meaning in shaping metaphorical meaning. One perspective holds that literal meaning actually contributes to the formation of metaphorical meaning (Grice, 1975; Searle, 1979; Weiland et al., 2013; Carapezza, 2019), while another viewpoint rejects the activation or involvement of literal meaning in this process. These proponents argue that metaphorical meaning is constructed either directly or in real time (Bezuidenhot, 2001; Recanati, 2004) or as an *ad hoc* concept (Carston, 2002; Sperber and Wilson, 2008).

As long as metaphor is treated and investigated as a natural kind, these three overarching questions around which Metaphor Studies revolve will remain unresolved antinomies, both theoretically and empirically. However, if we acknowledge the heterogeneity of the metaphorical phenomenon and abandon the Natural Kind Assumption (along with the claim to develop a comprehensive theory of metaphor that explains all its manifestations), we can ask whether they are in fact conflicting theories and conflicting data. Instead, we can consider whether the different answers to the three questions refer to different types of metaphor, and whether the different theories of metaphor are in fact incommensurable because they deal with phenomena of different nature.

Recently, some theoretical approaches, which we can call “hybrid”, have recognized the versatility of the metaphorical phenomenon noting precisely that the different theories of metaphor are not only opposed and contradictory, but also refer to different types of metaphor, to different phenomena that fall under the umbrella of metaphor.

Giora (2003), for example, through the concept of “salience” distinguishes three types of metaphor, each corresponding to different modes of processing. Giora defines salient meaning as “the meaning that first comes to mind” and distinguishes between: (1) Idioms and catachresis in which the salient meaning coincides with the figurative meaning and is processed before the literal meaning. For example, in the idiom “legs of the table” the figurative meaning is the one that comes to mind first, and an inferential effort is needed to derive the literal meaning from it, which refers to human legs. (2) In conventional metaphors, such as “Ludwig is a lion”, both the literal meaning (Ludwig is literally a lion) and the metaphorical meaning (Ludwig is very brave) are equally salient and are activated in parallel. (3) In novel metaphors, the most salient meaning corresponds to the literal meaning, which is activated first and, according to Grice (1975) and Searle (1979), is rejected and implicitly reinterpreted. Thus, according to Giora metaphor comprehension is based on the degree of salience of a lexical item. This degree determines whether sequential or parallel processes occur, so that different linguistic expressions arranged on a salience scale entail different processes.

This is also true for Steen (2008, 2023), who distinguishes between deliberate metaphors and non-deliberate metaphors – that is, linguistic expressions that are produced with the intention of creating a metaphor and require special attention (Cuccio, 2018), and linguistic expressions that only have a metaphorical past but are no longer processed as such (Lakoff and Johnson, 1980). Similarly, Green (2017) and Carston (2018), following Davies (1982), note that the contrast between Proposition Theories of metaphor and Image Theories of metaphor is not simply a matter of opinion, but each theory addresses different types of metaphor. In this context, Green distinguishes between Image Demanding Metaphors and Image Permitting Metaphors – metaphors where the activation of imagery is possible but not necessary (such as conventional metaphors, e.g., “Ludwig is a lion”) and metaphors where the activation of imagery is necessary (such as novel metaphors, e.g., “The snow is a winter closet”). According to Green, the former can be well described by Proposition Theories of metaphor, while the latter can be better described by Image Theories of metaphor. In parallel, Carston (2010, 2018) distinguishes between local metaphors, such as “Ludwig is a lion”, which require a simple contextual modulation of meaning (and thus require neither the activation of literal meaning nor mental imagery) and whose processing is well described by Proposition Theories of metaphor, and extended and poetic metaphors, better described by Image Theories of metaphor, such as the verses of *The Lovesong of Alfred Prufrock* by T.S. Elliot, which we present here:

The yellow fog that rubs its back upon the window-panes, The yellow smoke that rubs its muzzle on the window-panes, Licked its tongue into the corners of the evening, Lingered upon the pools that stand in drains, Let fall upon its back the soot that falls from chimneys, Slipped by the terrace, made a sudden leap, And seeing that it was a soft October night, Curled once about the house and fell asleep.

With this type of metaphor, it is necessary to retain the literal meaning, subject it to slow and reflective inferences, and then derive the metaphorical meaning. In this process, a mental image can emerge that emphasizes the content of metaphors and makes it easier to remember, even if it remains an epiphenomenon of the deeper pragmatic and propositional processes.

In addition, another dual theory of metaphor is proposed by Canal et al. (2022), which distinguishes between physical and mental metaphors: while physical metaphors require inferences about physical attributes of the metaphor topic (e.g., “ballerinas are butterflies”), mental metaphors require inferences about psychological attributes of the metaphor topic. These two types of metaphors appear to require different processing modes based on a different role played by the Theory of Mind.

Although these approaches emphasize the heterogeneity of the metaphorical phenomenon and propose broader hypotheses about metaphor, they nevertheless do not escape our antinomies. For although they recognize the versatility of the metaphorical phenomenon, they are based on a conception of metaphor that rests on the syntactic-semantic structures of the utterance. Moreover, also in these approaches, metaphor is seen as something that is “naturally” present in language, a linguistic expression considered metaphorical regardless of its function, occurrence, context and user. These scholars are looking for an internal feature of language, whereas the focus should be on how the language is used. They are looking for internal, structural features that make a linguistic expression a metaphor, whereas it is probably the contexts of use that make a linguistic expression a metaphor and determine what we do with that metaphor – whether a linguistic expression is a metaphor with a metaphorical meaning or whether it is an unfamiliar noise, whether it requires the lingering of the literal meaning or it is processed directly as an *ad hoc* concept, whether it is a figure of speech or plays a more significant role in our cognition.

So, these hybrid approaches still do not resolve the antinomies. They do not resolve them because, while recognizing the multifaceted nature of the metaphorical phenomenon, they continue to regard metaphor as a natural kind – a notion corresponding to a bunch of phenomena that can be defined as “metaphorical” and share a common essence. Moreover, these approaches remain bound to certain theoretical assumptions and thus offer a limited and highly theory-bound view of metaphor, losing sight of fundamental aspects of the phenomenon.

### 3.2 The heterogenic view of metaphor

In the following discussion we will present evidence that metaphor is not a homogeneous phenomenon and consequently cannot be considered a natural kind.

As can be seen from the first antinomy – is metaphor a matter of style or thought? – the term “metaphor” means in different theories:

– A cognitive mechanism that enables us to “see the similarities in the dissimilar” and thus create new knowledge (Vico, 1744/1977; Nietzsche, 1873; Blumenberg, 1960/2010; Aristotle, 2004).
– A conceptual structure that organizes our cognitive system by structuring abstract concepts through concrete concepts (Lakoff and Johnson, 1980; Gallese and Lakoff, 2005; Lakoff, 2009).
– A linguistic mechanism that merges terms belonging to different semantic domains (Richards, 1936/95; Black, 1962; Ricoeur, 1975; Kittay, 1987; Camp, 2003) or the realization of such a mechanism in the form of an utterance (Sperber and Wilson, 2008; Carston, 2010, 2018).

It is evident that these three meanings of “metaphor” have no common characteristics and do not refer to a unified category of phenomena. Instead, they denote three different levels of description that refer to three different types of phenomena that are to some extent related but not comparable. If we leave aside the first two meanings of the term “metaphor” and focus only on the third meaning, which refers to metaphor as a type of utterance, we also find that generalizations applicable to all forms and uses of metaphor are not possible.

**FIGURE 1:**
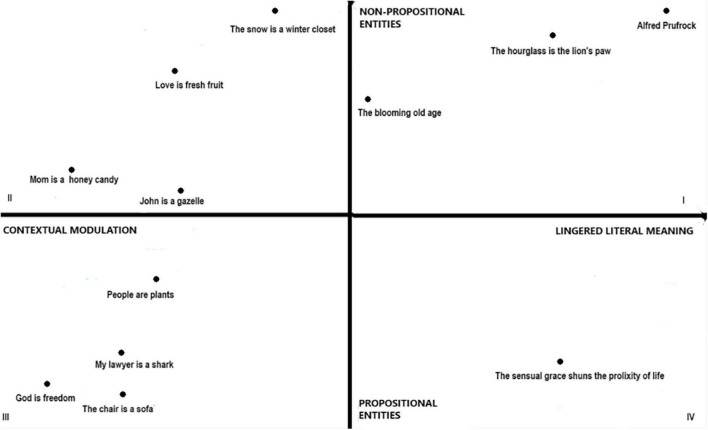
Orthogonal chart with different kind of “metaphors”.

If we examine the two parameters mentioned in the previous paragraph devoted to the second and third antinomies — namely, the reliance on literal meaning and the use of non-propositional entities in the interpretation of a metaphor — it becomes possible to recognize different metaphorical expressions that can be arranged in an orthogonal diagram. On the horizontal axis we position the spectrum of contextual modulation and persistence of literal meaning, while on the vertical axis we denote the involvement of a non-propositional dimension (Figure 1).

The x-axis varies according to the use of the literal meaning: on the left side we find metaphors that require contextual modulation of the meaning – that is, the derivation of an *ad hoc* concept – while on the right side we find metaphors that activate the literal meaning. The y-axis, on the other hand, varies depending on the activation of mental imagery or other related non-propositional entities (such as perceptual, emotional and sensorimotor mechanisms): at the top we find metaphors that activate mental imagery (or, in general non-propositional entities), while at the bottom we find metaphors that do not activate mental imagery (Garello and Carapezza, 2023). The position of the metaphor on these coordinates could vary as a function of the vividness of mental imagery and the strength of the non-propositional entities associated, which could depend on a number of factors: (1) the concreteness of the target and vehicle of a metaphor (Gleason, 2009): metaphors consisting of concrete targets and vehicles (“the snow is a winter closet”) or of abstract targets and concrete vehicles (“love is fresh fruit”) will presumably be located higher than metaphors consisting of abstract targets and vehicles (“God is freedom”). (2) The specificity of the target and vehicle of a metaphor (Bolognesi and Caselli, 2022): metaphors consisting of general targets and vehicles (people are plants) will presumably be located lower than metaphors consisting of specific targets and vehicles (mom is a honey candy). (3) The animacy of the target and vehicle of a metaphor (Cuccio et al., 2013): metaphors that consist of animate targets and vehicles (John is a gazelle) or inanimate targets and animate vehicles (the blooming old age) will presumably be located higher than metaphors that consist of inanimate targets and vehicles (the chair is a sofa). (4) The conventionality of the metaphor (Green, 2017): conventional metaphors (my lawyer is a shark) will be located lower than novel metaphors (love is fresh fruit). (5) The type of meaning we want to convey: if a concrete, animate, or specific meaning is intended, our metaphor will presumably be located higher than a metaphorical utterance with an abstract, inanimate, or general meaning. Thus, if “John is a lion” is uttered with the intention of conveying that “John is brave,” conveying an abstract meaning [as defined by Canal et al. (2022)] will be located lower than the analogous use to convey that “John has a thick mane,” which conveys a physical and concrete meaning (see Garello, 2024). If we stick to the conventional numbering of quadrants in a Cartesian diagram, we can find: (1) In the first quadrant extended metaphors (e.g., the fog described in “The Love Song of J. Alfred Prufrock”) or highly poetic metaphors where literal meaning and a non-propositional dimension must be considered. (2) In the second quadrant are metaphors that require pragmatic modulation in the explicature of the utterance and for which the formation of mental imagery may be necessary (if the metaphor is novel and/or characterized by a concrete, animate, or specific vehicle and/or conveys a concrete or animate or specific meaning) or otherwise unnecessary. (3) In the third quadrant are strongly conventional metaphors and catachresis which, being understood as phenomena of polysemy, require the derivation of an *ad hoc* concept and do not activate a non-propositional dimension. Or again, metaphors that have both an abstract or general or inanimate target and vehicle (e.g., “God is truth”, “Justice is freedom”). (4) Finally, in the fourth quadrant are metaphors that require the lingering of the literal meaning but do not activate mental imagery, such as “the sensual grace shuns the prolixity of life”.

### 3.3 Metaphor is not a natural kind

Based on the observation that the contradictory answers to our antinomies refer to different phenomena and thus highlighting the heterogeneity of the term “metaphor”, we can now question the three premises on which the Natural Kind Assumption (NKA) is based and that we recall here:

(1) Metaphor, despite their differences, share non-accidental common features. “Metaphor” represents a class of homogeneous phenomena with common properties.
(2) The presence of these properties is the essential precondition for defining an element as metaphorical.
(3) Metaphors have specific properties that distinguish them from other types of phenomena.

Against these premises, we claim that:

(1a) Metaphors do not represent a class of homogeneous phenomena with common properties.
(2a) If present, the shared properties between different types of metaphors are not essential to define an element as metaphorical.
(3a) Metaphors do not have specific properties that distinguish them from other types of phenomena.

We begin by challenging the first two premises of the Natural Kind Assumption, against which we argue that: (1) metaphor, in light of the Heterogenic view, does not represent a class of homogeneous phenomena with common properties, as it is shown by the schematic representation made in the previous section. (2) If present, the shared properties between different types of metaphors are not essential to define an element as metaphorical. In other words, it seems that there are many different kinds of metaphorical utterances that have little in common in terms of their syntactic, semantic and pragmatic features, as well as the way they are processed and the functions they serve in a given context. Any similarities that exist between these types of metaphors are better explained by the Wittgensteinian notion of “family resemblance” than by a common mechanism.

Instead of producing something common to all that we call language, I am saying that these phenomena have no one thing in common which makes us use the same word for all but that they are related to one another in many different ways. And it is because of this relationship, or these relationships, that we call them all language (Wittgenstein, 1953, § 65).

Let us consider the processes we define as “games”: we have board games, chess, card games, ball games, sports competitions and so on. If we try to find a common element between these “games”, we will not find it. We will see similarities and relationships but no element that is constantly present in all types of games.

Consider for example the proceedings that we call “games”. I mean board-games, card-games, ball-games, Olympic games and so on. What is common to them all? Don’t say: “There must be something common, or they would not be called “games” but look and see whether there is anything common to all. For if you look at them you will not see something that is common to all but similarities, relationships and a whole series of them at that. To repeat: *don’t think but look!* […] Can see how similarities crop up and disappear. And the result of this examination is: *we see a complicated network of similarities overlapping and criss-crossing*: sometimes overall similarities, sometimes similarities of detail (Wittgenstein, 1953, § 66 – *italics ours*).

With the words “don’t think, but look,” Wittgenstein challenges us to adopt an approach to understanding language and reality that emphasizes the direct observation of phenomena over abstract conceptual analysis. We observe phenomena without becoming too entangled in conceptual considerations and, in our case, in metalinguistic categories. What is observed in games, as in the various kinds of “metaphors”, are not unique elements, or “essences” but a “complicated network of overlapping and intersecting similarities” (Wittgenstein, 1953, § 66). We call something a “game” or, in our case a “metaphor”, because it has a kinship with something that has been called that way before and thus acquires an indirect kinship with other things so named. This does not mean, however, that the game, or metaphor, is a homogeneous class of phenomena with similar and specific properties. Rather, what we call “metaphor” is neither *a priori* limited, nor *a priori* limitable. The similarities between different kinds of metaphors emerge and disappear as a function of contexts and are not the result of an underlying and common mechanism.

Finally, we come to the criticism of the third premise of the NK assumption. Against it we claim that metaphors don’t have specific properties that distinguish them from other types of phenomena. The different types of metaphors that we have grouped and systematized in the model in the previous section do not seem to have common properties that are due to a causal mechanism (according to the definition provided by Boyd (1990, 1991). Therefore, we can now try to understand whether the different types of metaphors have features specific to each quadrant or whether such features can be found in other utterances and uses of language. It seems to us that even in this case, it is not possible to identify unique and specific features for each type of metaphor.

Specifically, in the first quadrant, where we found extended and imagistic metaphors (such as *The Love Song* by Alfred Prufrock), we could also find haikus or imagistic descriptions that also require the use of literal meaning and in which the imagistic dimension seems to be particularly vivid and conscious. We think, for example, of the short Japanese composition by Mizuta Masahide (1657- 1723): “Barn’s burnt down/now I can see the moon.” Or the composition by Yosa Buson (1716-1784): “Such a moon/the thief pauses to sing.” In these haikus, the use of literal meaning is essential to the construction of haiku’s meaning and indeed it is precisely on the basis of literal meaning that mental imagery is evoked and could plausibly play a central role in constructing the meaning of the composition. The conceptual density of haiku might be given precisely by a process of mutual adjustment between the explicit and literal level and the implicit level mediated by the imagery – as seems to happen in the extended metaphors we have placed in the first quadrant. Therefore, metaphors found in the first quadrant, i.e., extended metaphors characterized by an imagistic dimension in which metaphorical meaning is constructed through the use of literal meaning and the relationship between literal and metaphorical meaning is mediated through mental imagery, seem to share the same processes of meaning construction common to other linguistic uses, such as haiku and, in general, literal imagistic descriptions.

Similarly, the metaphors found in the second and third quadrants, i.e., “local” metaphors, where the metaphorical meaning is constructed directly in the explicature of the utterance by deriving an *ad hoc* concept and may or may not activate mental imagery depending on the characteristics of the vehicle and topic, seem to undergo the same processes of meaning construction found in other more or less novel linguistic uses: the processes of narrowing and broadening by which we construct *ad hoc* concepts are at work in much of our conversation every day and non-propositional entities play a more or less prominent role depending on the contextual and linguistic features of the conversational exchange.

**FIGURE 2:**
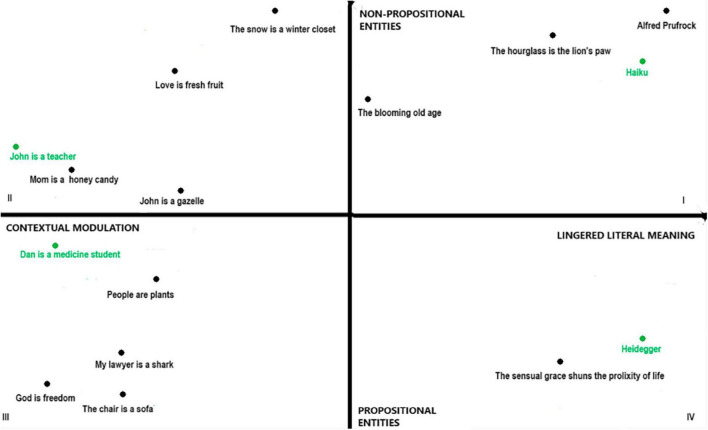
Orthogonal chart: different kinds of “metaphor” and other kinds of utterance in relation.

The fourth quadrant, in which we find complex or extended metaphors with abstract targets and vehicles, can therefore be shared with other highly abstract but not strictly metaphorical uses of language, such as certain forms of philosophical abstraction, as in the works of Hegel, Fichte, or Heidegger. In Heidegger (2019, 90), for example, we find the utterance, “The metaphysical representation owes its view to the light of being. The light, or rather what such thought experiences as light, does not itself fall within the view of this thought because it represents being only ever by looking at beings” (*our translation*) (Figure 2).

At the procedural level, then, metaphors do not seem to have any special features reserved for them: the identification of the four types of metaphors and the four ways of constructing metaphorical meaning does not imply the recognition of a special status for metaphors and indeed each “way” is shared by a type of metaphor with other linguistic uses.

The three premises on which the Natural Kind Assumption rested thus seem to be crumbling: metaphors are not a unified and clearly definable phenomenon but rather a flexible, complex and multilayered theoretical construct. Thus, there is no a fixed, theory-independent class of objects in the world called “metaphor” and there is nothing consistent with the intuition that there is a common and specific set of properties that uniquely identify a class of objects in terms of metaphor. In this way our antinomies dissolve: it is not possible to give an unambiguous answer to the questions from which the antinomies of metaphor arise, because there is no clearly and unambiguously definable class of objects in the world called “metaphor”. Rather, what a metaphor is and how it works depends on our beliefs and theories about how “language” works in general. Metaphor, therefore, turns out to be an unstable theoretical construct to which only provisional answers can be given. When we move from the level of the functioning of metaphor to the level of formalization in search of a definition of “metaphor”, we find that it is only provisional and definable within the framework of a theory. Thus, a definition of metaphor seems to depend on the theory of language on which it is based. Namely, the definition of metaphor is inherently tied to the theoretical underpinnings of the language framework within which it is situated. Different theories offer different lenses through which metaphor can be understood. For this reason, there are not “false theories” of metaphor but only “partial theories of metaphor” and each one grasps an aspect of it. In a nutshell, metaphor is not a natural kind.

## 4 Eliminativism or preservation? The future of metaphor studies

Therefore, metaphor seems not to be a natural kind but a complex and multifaceted theory-dependent philosophical notion. Consequently, the term “metaphor” seems not to be a natural kind term. Scholars who talk about “metaphor” often refer to very different phenomena that might even turn out to be incommensurable. The sense of metaphor referred to by Black, Nietzche, Lakoff and Johnson seems to be very different to the sense of metaphor found in Davidson and Rorty or in Grice and Sperber and Wilson.

The term “metaphor” and the phenomena to which it refers differ so much within the various theories of metaphor that it is not possible simply to compare or evaluate these various uses by appealing to supposedly theory-neutral observations. On this basis, we can ask what theoretical implications arise for studies of metaphor. We believe that this recognition can lead to two opposing attitudes. On the one hand, an attitude in which, since metaphor is not a natural kind and the meanings of the term are often incomparable, the study of metaphor should be abandoned. Metaphor is thus seen as a superfluous theoretical category and, to follow Occam’s principle, we may choose not to include it in the Olympus of topics that can be studied. The opposite stance, to which we fully subscribe, recognizes that metaphor is not a natural kind and it acknowledges the challenges of Metaphor Studies, but this does not lead us to deny the relevance of metaphor in language studies.

This way seems to lead neither to antinomies nor to the abandonment of millennia-old studies on metaphor. It is a matter of recognizing minimal, general operations, not just those peculiar to metaphor, which serve as a hinge for all meanings of the term, without resorting to an assumed “essence” of metaphor: the different meanings of the term “metaphor” would be linked by “family resemblances” that are not unique to metaphor but are also common to other linguistic-cognitive processes. Therefore, while it is not possible to identify unifying features and processes between different types of metaphor, it is possible to recognize that they are the result of generic mechanisms of linguistic-cognitive creativity that create new knowledge and represent things differently by connecting distant elements – such as the processes of “bisociation of ideas” (Koestler, 1964) or “condensation” (Freud, 1899) or the more general processes of semantic shift, in which two objects or ideas that were not previously associated are connected and concentrated in a single representation with a variable degree of intensity during the history of a word (Bowdle and Gentner, 2005). These minimal and generic mechanisms do not constitute the essence of metaphor for two reasons:

(I) They are not particular to metaphor, but are common to other human creative processes, such as scientific discovery, theorizing, art production, and dreaming.
(II) Because of their universality, these mechanisms do not identify a homogeneous group of linguistic and non-linguistic entities but manifest themselves differently in different products of human activity (utterances, scientific theories, works of art, etc.).

It is therefore not possible to find unifying elements among metaphors that identify stable structural features, apart from the very general operations related to human creativity that are also common to other linguistic-cognitive processes. However, it is possible to recognize “family resemblances”, where an expression is defined as a “metaphor” because it has a direct kinship with something that was previously called a “metaphor”, thus extending our concept of “metaphor” and continuously overlapping with other meanings of the term.

This view seems to be close to the Aristotelian view of metaphor in the *Rhetoric*: Aristotle, in fact, used the term *metaphorà* to refer not only to metaphors in the narrow sense in which they are understood today, but to a process that produces “brilliant expressions” of various kind and, more generally, to figurative language as a whole, which is the result of the speaker’s ability to “bring things before the eyes” of the listener through the use of such linguistic expressions.

With this solution, although we deny that metaphor is a natural kind and that there is therefore a unique and homogeneous phenomenon defined as “metaphor”, we recognize the existence of different types of metaphor that are not reducible to each other and are the result of general processes of human creativity that allow us to productively connect different domains. However, these mechanisms are not specific to metaphors. Among the different types of metaphor, it is therefore not possible to identify unique and universal characteristics, but “family resemblances” or “symptoms” that make an utterance metaphorical or not on the basis of several factors. Just as with all other uses of language.

Moreover, rather than abandoning this complex field of research, we believe that the recognition that metaphor is not a natural kind but a complex philosophical notion theory-dependent can actually enrich and clarify this field of research. In fact, from this observation comes two needs: first of all, it poses the problem of finding empirical approaches to the study of a notion that is not a natural kind but may have interesting empirical applications and implications. Secondly, it highlights the need to make the different theories of metaphor comparable to each other. Embracing the complexity of metaphor enriches our understanding of language and paves the way for a more nuanced and comprehensive exploration of this intricate facet of human form of life.

## 5 Conclusion

The productive vitality of metaphor research of the last 50 years has led to considerable confusion in this area of research. The term “metaphor” has become so layered over the centuries that it has come to denote an indefinite variety of notions and objects, many of which differ greatly from one another. The question “what is a metaphor?” therefore has become “what are we talking about when we talk about metaphors?”. In trying to provide an album to help navigate the diverse and numerous theories on this topic of importance to philosophy of language, linguistics and cognitive science, we have identified three questions around which the discussion of metaphor revolves: 1) is metaphor a matter of style or of thought? 2) What is and how is the meaning of a metaphor constructed? 3) What is the relationship between literal and metaphorical meaning?

We have found that it is not possible to provide a clear and definitive answer to this questions that represent what we have called “the antinomies of metaphor.” These antinomies are linked to a common assumption that run through Metaphor Studies, namely the assumption for which metaphor is a natural kind.

In contrast, this paper challenges this Natural Kind Assumption and argues that metaphors do not constitute a natural kind but rather a complex, theory-dependent philosophical notion. This departure from the traditional view prompts a reconsideration of the implications for a theory of language, particularly in the context of Metaphor Studies. In particular, the recognition that metaphor is not a natural kind does not lead to the rejection of Metaphor Studies, but rather underlines the need to reconsider theoretical and empirical approaches that address the poliedric and multi-layered nature of a philosophical notion that is as interesting as it is complex.

## Author contributions

SG: Conceptualization, Investigation, Writing – original draft. MC: Conceptualization, Investigation, Writing – review and editing, Funding acquisition, Project administration, Resources, Supervision.
